# Three New Alien Taxa for Europe and a Chorological Update on the Alien Vascular Flora of Calabria (Southern Italy)

**DOI:** 10.3390/plants9091181

**Published:** 2020-09-11

**Authors:** Valentina Lucia Astrid Laface, Carmelo Maria Musarella, Ana Cano Ortiz, Ricardo Quinto Canas, Serafino Cannavò, Giovanni Spampinato

**Affiliations:** 1 Department of AGRARIA, Mediterranean University of Reggio Calabria, Loc. Feo di Vito snc, 89122 Reggio Calabria, Italy; vla.laface@unirc.it (V.L.A.L.); serafino.cannavo@unirc.it (S.C.); gspampinato@unirc.it (G.S.); 2 Department of Animal and Plant Biology and Ecology, Section of Botany, University of Jaén, 23071 Jaén, Spain; anacanor@hotmail.com; 3 Faculty of Sciences and Technology, University of Algarve, Campus de Gambelas, 8005-139 Faro, Portugal; rjcanas@ualg.pt; 4 Centre of Marine Sciences (CCMAR), University of Algarve, Campus de Gambelas, 8005-139 Faro, Portugal

**Keywords:** biodiversity, check-list, exotic plant, herbarium specimens, invasiveness, new floristic records, plant diversity

## Abstract

Knowledge on alien species is needed nowadays to protect natural habitats and prevent ecological damage. The presence of new alien plant species in Italy is increasing every day. Calabria, its southernmost region, is not yet well known with regard to this aspect. Thanks to fieldwork, sampling, and observing many exotic plants in Calabria, here, we report new data on 34 alien taxa. In particular, we found three new taxa for Europe (*Cascabela thevetia*, *Ipomoea setosa* subsp. *pavonii*, and *Tecoma stans*), three new for Italy (*Brugmansia aurea*, *Narcissus* ‘*Cotinga*’, and *Narcissus* ‘*Erlicheer*’), one new one for the Italian Peninsula (*Luffa aegyptiaca*), and 21 new taxa for Calabria (*Allium cepa*, *Asparagus setaceus*, *Bassia scoparia*, *Beta vulgaris* subsp. *vulgaris*, *Bidens formosa*, *Casuarina equisetifolia*, *Cedrus atlantica*, *Chlorophytum comosum*, *Cucurbita maxima* subsp. *maxima*, *Dolichandra unguis-cati*, *Fagopyrum esculentum*, *Freesia alba*, *Juglans regia*, *Kalanchoë delagoënsis*, *Passiflora caerulea*, *Portulaca grandiflora*, *Prunus armeniaca*, *Prunus dulcis*, *Solanum tuberosum*, *Tradescantia sillamontana*, and *Washingtonia filifera*). Furthermore, we provide the first geolocalized record of *Araujia sericifera*, the confirmation of *Oxalis stricta*, and propose a change of status for four taxa (*Cenchrus setaceus*, *Salpichroa origanifolia*, *Sesbania punicea*, and *Nothoscordum gracile*) for Calabria. The updated knowledge on the presence of new alien species in Calabria, in Italy and in Europe could allow for the prevention of other new entries and to eliminate this potential ecological threat to natural habitats.

## 1. Introduction

The increase in alien plant invasion represents a very significant ecological problem for native flora [1]. This produces several impacts around the world such as the reduction of native and endemic species and habitats, but also damage to socio-economic and human health [2,3,4,5,6]. For example, *R. pseudocacia* L. has several effects on the endangered lichen species *Lobaria pulmonaria* (L.) Hoffm. (loss of diversity, among others) that are also due to climate change [7,8,9]. Thanks to a recent work, several invasive alien species were assessed in Italian regions [10]: in Toscana, *Carpobrotus acinaciformis* (L.) L. Bolus produces a reduction in native biodiversity and habitat degradation in the habitat of 1240, whereas in Lombardia, *Lagarosiphon major* (Ridl.) Moss produces primary production alteration and modification of natural benthic communities in the habitats of 3140 and 3150, respectively.

Although Calabria has been the object of interest by numerous scholars since 1800 [11,12,13,14,15], only in recent decades has there been a significant increase in botanical studies that have affected its territory in various investigation fields: such as floristic [16,17,18,19,20,21,22], phytosociological [23,24,25,26,27,28], ethnobotanical [29,30,31,32,33,34,35], and many others [36,37,38,39,40,41,42]. However, these works only in part provided data on the alien flora present in the Calabrian territory.

According to Bartolucci et al. [43], Calabrian vascular flora amounts to 2799 taxa including native, cryptogenic, and alien taxa. As for these latter, Galasso et al. [1] recorded, for this region, “only” 267 alien taxa; therefore, Calabria seems to be one of the Italian regions with the fewest exotic taxa in a spontaneous state! According to Musarella et al. [44], this is probably due to a lack of knowledge of vascular flora in the most anthropized areas. However, many contributions were later added to [1] and have increased the knowledge on the invasive alien flora in Calabria [44,45,46,47,48,49,50,51,52]. A recent work estimates that Calabrian alien vascular flora amounts to 336 taxa [53]. Subsequently, another eight taxa have been reported as new to Calabria by Galasso et al. [54].

**Figure 1:**
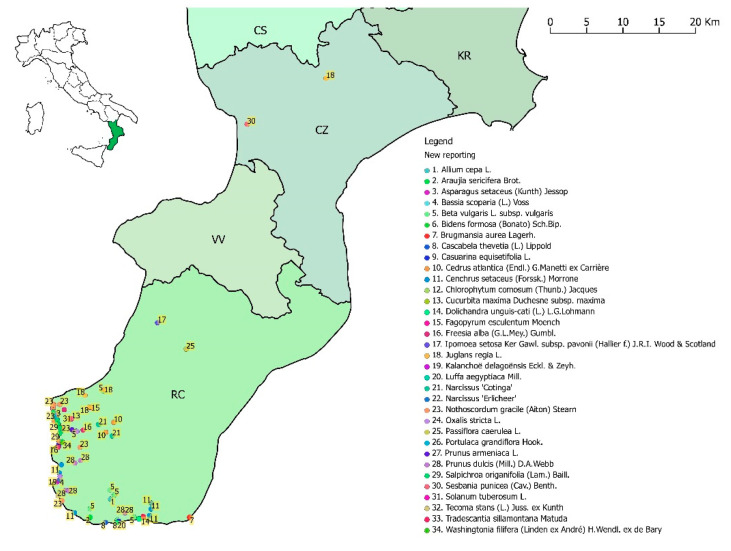
Distribution of the 34 alien taxa recorded for the flora of Calabria (Southern Italy, Europe). Calabrian provinces: CS = Cosenza, CZ = Catanzaro, KR = Crotone, RC = Reggio Calabria, VV = Vibo Valentia.

This paper aims to document new records (collected specimens and observations) of alien plant taxa for Calabria (Figure 1). For each taxon, relevant information about the ecology and distribution and a careful assessment of the naturalization status is also provided.

## 2. Results

**Figure 2:**
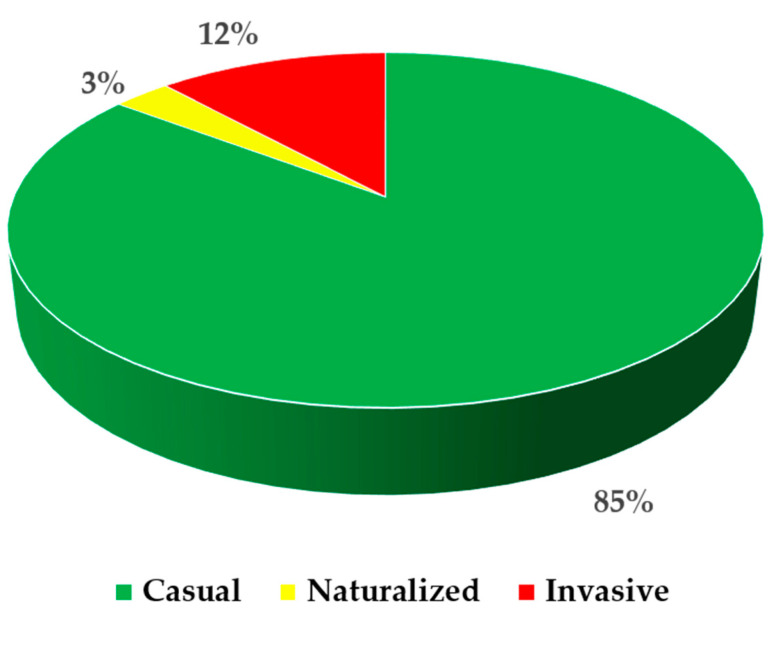
Current status of invasiveness of the 34 alien taxa recorded for the flora of Calabria (Southern Italy, Europe).

In this research work, we report 34 alien taxa recorded in several places of Calabria with different status of invasiveness (Figure 2; Table 1; Appendix A). Among these, three taxa are new for Europe (*Cascabela thevetia*, *Ipomoea setosa* subsp. *pavonii*, and *Tecoma stans*), three are new for Italy (*Brugmansia aurea, Narcissus* ‘*Cotinga*’, and *Narcissus* ‘*Erlicheer*’), one is new for the Italian Peninsula (*Luffa aegyptiaca*), and 21 are new for Calabria (*Allium cepa*, *Asparagus setaceus*, *Bassia scoparia*, *Beta vulgaris* subsp. *vulgaris*, *Bidens formosa*, *Casuarina equisetifolia*, *Cedrus atlantica*, *Chlorophytum comosum*, *Cucurbita maxima* subsp. *maxima*, *Dolichandra unguis-cati*, *Fagopyrum esculentum*, *Freesia alba*, *Juglans regia*, *Kalanchoë delagoënsis*, *Passiflora caerulea*, *Portulaca grandiflora*, *Prunus armeniaca*, *Prunus dulcis*, *Solanum tuberosum*, *Tradescantia sillamontana*, and *Washingtonia filifera*). Furthermore, we provide, for Calabria, the first geolocalized record of *Araujia sericifera*, the confirmation of *Oxalis stricta*, and propose a change of status for *Cenchrus setaceus*, *Salpichroa origanifolia*, and *Sesbania punicea* from naturalized to invasive, and for *Nothoscordum gracile* from casual to invasive. Some taxa were found in only one place, while others in several places, for a total of 118 records (Table S1).

In particular, as below discussed and already confirmed by Spampinato et al. [50], *Cenchrus setaceus*, among others, showed a high degree of invasion in natural habitats. Other taxa have also been found in protected sites falling within the Natura 2000 network: *Beta vulgaris* subsp. *vulgaris*, *Cedrus atlantica*, *Fagopyrum esculentum*, *Freesia alba*, *Juglans regia*, and *Nothoscordum gracile*.

## 3. Discussion

The continuous and constant increase in a short time of new records of alien taxa in the Calabrian territory, and generally in Italy and Europe, represents a worrying threat for the autochthonous biodiversity and a clear sign of the ongoing climate change [55].

As shown in Figure 1, most of the new reports of alien taxa occurred within inhabited areas; these have a high risk of future invasion of natural habitats [56]. A total of 85% of recorded taxa is “casual”, 3% “naturalized”, and 12% is “invasive”. This last percentage of invasive taxa comes from a change of status that we propose in the present work, which means that several taxa continue to expand their range of distribution, representing a constant threat for native species and habitats.

**Figure 3:**
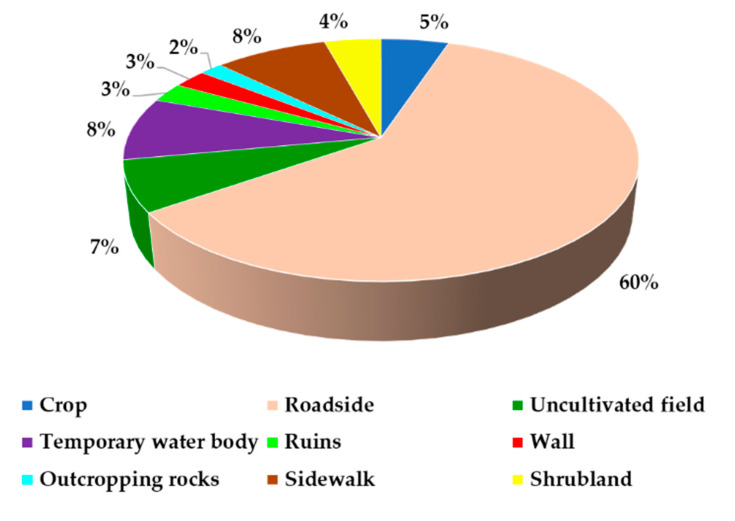
Habitats of the 34 alien taxa recorded for the flora of Calabria (Southern Italy, Europe).

Several records have been found along roadsides (Figure 3). Indeed, roadsides are places where alien taxa can establish [57,58] and are a considerable way to invade other areas [59]. Obviously, roads were also the places traveled by us during our research as it is more common to find new alien taxa along them. This does not mean that other habitats are not good places for alien taxa: in fact, numerous taxa have been encountered in other habitats but not considered here because they have already been reported previously for Calabria. In this study, we found that, out of 118 records, 60% were found along roadsides (Figure 3)! Only 8% were found in sidewalks and in temporary water bodies such as drainage channels and *fiumare* (typical rivers of southern Italy with torrential and irregular regime.), 7% in uncultivated fields, 5% in crop habitats, and 12% in other habitats (outcropping rocks, walls, shrublands and ruins). More details are reported in Table 2.

We verified the distribution of taxa within the Natura 2000 network. Some taxa grew in network sites: *Beta vulgaris* subsp. *vulgaris*, *Cedrus atlantica*, *Fagopyrum esculentum*, *Freesia alba*, *Juglans regia*, and *Nothoscordum gracile* (Table 3). *B. vulgaris* subsp. *vulgaris* was found in SAC IT9350145 “Fiumara Amendolea (including Roghudi, Chorio, and Rota Greco)” and in SPA IT9350300 “Costa Viola”, while *C. atlantica*, *F. esculentum*, *F. alba*, *J. regia*, and *N. gracile* were only found in the SPA IT9350300 “Costa Viola”. If *C. atlantica*, *F. esculentum*, *F. alba*, and *J. regia* are considered as “casual”, *B. vulgaris* subsp. *vulgaris* tends to naturalize, and *N. gracile* becomes invasive in other sites where it has been found, also representing a potential threat for protected areas such as SPA “Costa Viola”.

Two species (*Araujia sericifera* and *Washingtonia filifera*) are recently included in a list of candidate species to be submitted to the national prioritization procedure for their inclusion in a national list according to Regulation (EU) No. 1143/2014 [62]. Therefore, knowledge of new sites of *A. sericifera* and the new record of *W. filifera* for Calabria are very important for their control because they have a detrimental impact on plant communities [63].

Among others, *Cenchrus setaceus* is rapidly spreading in the regional territory after its first report in 2007 [64]. Indeed, these authors reported *C. setaceus* (sub *Pennisetum setaceum*) for Calabria in two locations, both along communication routes, but at a high distance from each other: one along the A2 motorway between Rosarno and Gioia Tauro in the province of Reggio Calabria on the Thyerrenian side, and another along highway SS 106 Jonica near Cropani Marina in the province of Catanzaro on the Ionian side of the region. After several new records of this alien species for Calabria by Musarella et al. [44,50], new individuals have been recorded near the previously observed in Reggio Calabria where they have generated new tufts in the same point [“lungo Raccordo Autostradale 4 di Reggio Calabria prima della galleria Spirito Santo in direzione Sud (Reggio Calabria), roadside, 93 m s.l.m., 21.11.2018, 557933–4218164, obs. et det. V.L.A. Laface et C.M. Musarella”] and a few tens of meters from them [“lungo la SS 106 Jonica presso località Ravagnese (Reggio Calabria), roadside, 38 m s.l.m., 19.11.2018, 558138–4213357, leg. et det. C.M. Musarella (REGGIO)] [44]. According to Brundu [65], only an early detection and rapid eradication (EDRE) of *C. setaceus* can prevent its rapid spread, for this reason, its continuous monitoring is really important to prevent ecological damage.

**Figure A1:**
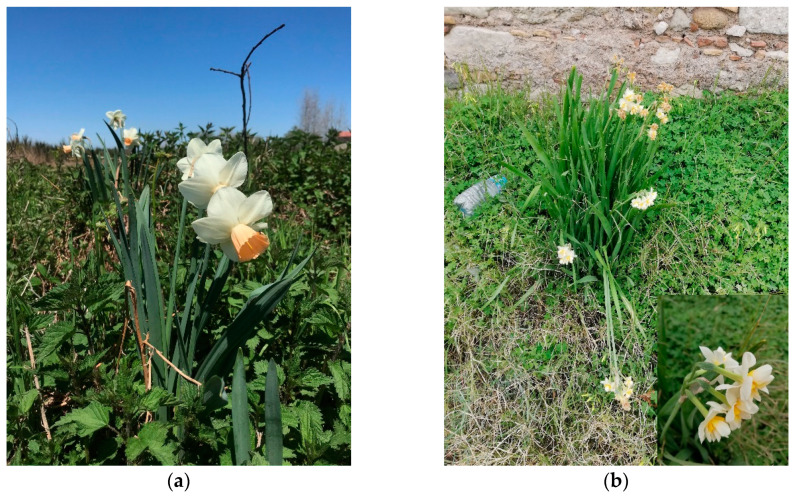
Two different cultivars of *Narcissus cv.* collected in the Reggio Calabria province. (**a**) *Narcissus ‘Cotinga’* in Piani di Reggio, in grazing-meadow (Photograph VLA Laface); (**b**) *Narcissus ‘Erlicheer’* in Pellaro, in a roadside along a wall (Photograph CM Musarella).

Two cultivars of *Narcissus* used as ornamentals were observed by the authors (Figure A1). Report no. 21 was recorded for two locations where it has multiplied over the years after the first observation in 2013 by Laface and report no. 22 was recorded for the first time at the beginning of 2020 by Musarella. It is likely that they escaped cultivation or were thrown among the waste material from nearby gardens. As to which cultivars they were, there are more than 27,000 daffodils of garden origin registered in the International Daffodil Register and Classified List in 2008 [66]. According to [66], the report no. 21, which has single flowers with solitary flowers, white reflexed perianth segments and an apricot-pink corona, belongs in Division 6 (Cyclamineus) and was recognized as *Narcissus ‘Cotinga’*. Report no. 22, which has double flowers with white perianth segments with a trace of yellow at the base, belongs to Division 4 (Double flowered) and is referred to as *Narcissus ‘Erlicheer’*. Regarding their invasiveness, it is important to consider that daffodils rarely spread rapidly and certainly the Division 4 cultivars are sterile, so cannot spread by seed. For this reason, we consider it to not have a detrimental impact on the environment. These are both first records for Italy.

## 4. Materials and Methods

This research was based on fieldwork carried out randomly and during a well-designed research field for the coordination of monitoring activities of natural and semi-natural habitats of the flora and fauna species of the Natura 2000 Network present in Calabria (see Funding section) from 2018 to 2020 as well as on herbaria and literature surveys. As part of this project, in fact, it was necessary to verify the conservation status of the habitats in accordance with the European Directive [60] as well as in relation to the possible threat or pressure exerted by an alien species on them. Fieldwork consisted of collecting samples of alien plants and identifying them in the laboratory. The collected specimens was stored dried in the herbarium of the Mediterranean University of Reggio Calabria (Italy) (REGGIO, acronym according to Thiers [67]). The investigated area was the whole of Calabria, but the new records concern only the central-southern part.

The taxa in the floristic list are arranged in alphabetical order (Appendix A). Nomenclature, taxa delimitation, and regional distribution are in accordance with Galasso et al. [1] and the following update [45,46,47,48,49,53]. The specimens were identified using Flora Europaea [68,69,70,71,72], Flora d’Italia [73,74,75,76,77], Flora of North America [78], Flora of China [79], and some monographic works [66,80,81,82]. The update distribution of the considered taxa was verified also using [83,84]. Life forms and native range were according to [73,74,75,76,77,85] and by on-field observation. Period of introduction of the taxa recorded were verified on “Portale della Flora d’Italia” [85].

For each taxa, we provide the following information: (1) accepted name; (2) basionym and most relevant synonyms; (3) plant family; (4) period of introduction (archeophyte, neophyte, or cryptogenic); (5) native range; (6) life form; (7) data record in Calabria, Italy, or Europe; (8) current invasiveness status for the region (according to Pyšek et al. [86]); (9) date of observation; (10) discovery localities (municipality, administrative province) (*exsiccata* and *observata*) with details on the location (in Italian, according to the information on the specimen labeldata); (11) terminology of Pyšek et al. [86]; (12) decimal degrees geographic coordinates (datum WGS84); (13) growth environment; (14) altitude (meters above sea level–m a.s.l.); (15) *legit* (or *observavit*) and *determinavit*; (16) herbarium where the specimen is stored; and (17) distribution and/or ecological notes when available. For some taxa not documented with a herbarium specimen, we report some pictures where available (Appendix B). All other plants pictures are reported in Appendix B.

## 5. Conclusions

Although there is adequate knowledge of the alien flora in Italy, not much is known about those in Calabria thus far. As before indicated, Calabrian alien vascular flora amounts to 344 taxa [53,54]. Thanks to this study, it was possible to increase this knowledge by reporting new data on 34 taxa: one (1) confirmation for Calabria, some new for Calabria (21), other new for the Italian Peninsula (1), three (3) for the whole of Italy, and three (3) new for the whole of Europe. Furthermore, a georeferenced location for *Araujia sericifera* (1) and the invasiveness status for four (4) species already known for the region are reported here for the first time. In total, 28 taxa are new to Calabria. Therefore, we can currently count 373 alien plant taxa for the whole of Calabria. This was possible thanks to the increasing attention to the exotic taxa introduced because, compared to the past, these taxa are invading more and more indigenous habitats, compromising their ecological balance and threatening the survival of native taxa. Works like these are very important because they provide the possibility of identifying a large number of new alien plant species that are spreading worldwide, allowing us to fill in the many gaps at the regional, national and global level.

Nonetheless, it is now clear that knowledge of native species is not enough for the protection of a specific territory. Of additional importance is the knowledge of alien species, their invasive potential, prevention in their introduction (both intentional and accidental), and their immediate eradication (i.e., *Cenchrus setaceus*, *Nothoscordum gracile*, *Salpichroa origanifolia*, and *Sesbania punicea*, which are spreading very rapidly as invasives). All these actions must be carried out by both the public and private institutions responsible for this, and most notably by citizens that can responsibly act autonomously in this sense. However, the ever-increasing diffusion of taxonomic, biological and ecological knowledge must form the basis for carrying out sustainable policies for the environment.

## Figures and Tables

**Table 1:** List of the 34 alien taxa recorded for the flora of Calabria (Southern Italy, Europe), with their eventual first record for Europe, first record for Italy, or confirmation for Calabria, and finally, the current status of invasiveness in the region and the previous one if changed.

ID	Taxon	First Record for Europe	First Record for Italy	First Record for Calabria	Confirmation for Calabria	Current Status	Previous Status
1	*Allium cepa* L.			X		C	
2	*Araujia sericifera* Brot.			G		C	
3	*Asparagus setaceus* (Kunth) Jessop			X		C	
4	*Bassia scoparia* (L.) Voss			X		C	
5	*Beta vulgaris* L. subsp. *vulgaris*			X		N	
6	*Bidens formosa* (Bonato) Sch. Bip.			X		C	
7	*Brugmansia aurea* Lagerh.		X			C	
8	*Cascabela thevetia* (L.) Lippold	X				C	
9	*Casuarina equisetifolia* L.			X		C	
10	*Cedrus atlantica* (Endl.) G. Manetti ex Carrière			X		C	
11	*Cenchrus setaceus* (Forssk.) Morrone					I	N
12	*Chlorophytum comosum* (Thunb.) Jacques			X		C	
13	*Cucurbita maxima* Duchesne subsp. *maxima*			X		C	
14	*Dolichandra unguis-cati* (L.) L.G. Lohmann			X		C	
15	*Fagopyrum esculentum* Moench			X		C	
16	*Freesia alba* (G.L. Mey.) Gumbl.			X		C	
17	*Ipomoea setosa* Ker Gawl. subsp. *pavonii* (Hallier f.) J.R.I. Wood & Scotland	X				C	
18	*Juglans regia* L.			X		C	
19	*Kalanchoë delagoënsis* Eckl. & Zeyh.			X		C	
20	*Luffa aegyptiaca* Mill.		IP	X		C	
21	*Narcissus ‘Cotinga’*		X			N	
22	*Narcissus ‘Erlicheer’*		X			C	
23	*Nothoscordum gracile* (Aiton) Stearn					I	C
24	*Oxalis stricta* L.				X	C	
25	*Passiflora caerulea* L.			X		C	
26	*Portulaca grandiflora* Hook.			X		C	
27	*Prunus armeniaca* L.			X		C	
28	*Prunus dulcis* (Mill.) D.A. Webb			X		C	
29	*Salpichroa origanifolia* (Lam.) Baill.					I	N
30	*Sesbania punicea* (Cav.) Benth.					I	N
31	*Solanum tuberosum* L.			X		C	
32	*Tecoma stans* (L.) Juss. ex Kunth	X				C	
33	*Tradescantia sillamontana* Matuda			X		C	
34	*Washingtonia filifera* (Linden ex André) H. Wendl. ex de Bary			X		C	

Notes: X = New or confirmed record; G = first Geolocalized record; IP = new record only for Italian Peninsula. Current and previous status of invasiveness: C = Casual; N = Naturalized; I = Invasive.

**Table 2:** Detailed list of the 34 alien taxa recorded to Calabria (Southern Italy, Europe) including family, period of introduction, native range, life form, habitat, and number (No.) of records for each taxon.

ID	Taxon	Family	Period of Introduction	Native Range	Life Form	Habitat	No. of Records
1	*Allium cepa* L.	Amaryllidaceae	Archeophyte	Culton	G bulb	Crop	1
2	*Araujia sericifera* Brot.	Apocynaceae	Neophyte	Southern America	P lian	Roadside	2
3	*Asparagus setaceus* (Kunth) Jessop	Asparagaceae	Neophyte	Southern Africa	P lian	Uncultivated field	5
4	*Bassia scoparia* (L.) Voss	Chenopodiaceae	Neophyte	Central Asia	T scap	Roadside	1
5	*Beta vulgaris* L. subsp. *vulgaris*	Chenopodiaceae	Archeophyte	Culton	H scap	Crop/Uncultivated field/Roadside	7
6	*Bidens formosa* (Bonato) Sch. Bip.	Asteraceae	Neophyte	Northern and Central America	T scap	Roadside	2
7	*Brugmansia aurea* Lagerh.	Solanaceae	Neophyte	Ecuador	NP	Roadside	1
8	*Cascabela thevetia* (L.) Lippold	Apocynaceae	Neophyte	Central and Southern America	Ch frut	Temporary water body	2
9	*Casuarina equisetifolia* L.	Casuarinaceae	Neophyte	Australia	P scap	Wall	2
10	*Cedrus atlantica* (Endl.) G. Manetti ex Carrière	Pinaceae	Neophyte	Northern Africa	P scap	Shrubland	2
11	*Cenchrus setaceus* (Forssk.) Morrone	Poaceae	Neophyte	Northern and Eastern Africa and Arabian Peninsula	H caesp	Roadside	6
12	*Chlorophytum comosum* (Thunb.) Jacques	Asparagaceae	Neophyte	Southern Africa	H scap	Temporary water body	2
13	*Cucurbita maxima* Duchesne subsp. *maxima*	Cucurbitaceae	Neophyte	Culton	T scap	Temporary water body/Roadside	2
14	*Dolichandra unguis-cati* (L.) L.G. Lohmann	Bignoniaceae	Neophyte	Southern America	P lian	Temporary water body/Ruins	3
15	*Fagopyrum esculentum* Moench	Polygonaceae	Neophyte	Asia	T scap	Roadside	1
16	*Freesia alba* (G.L. Mey.) Gumbl.	Iridaceae	Neophyte	Southern Africa	G bulb	Roadside/Ruins/Wall	3
17	*Ipomoea setosa* Ker Gawl. subsp. *pavonii* (Hallier f.) J.R.I. Wood & Scotland	Convolvulaceae	Neophyte	Southern America	G bulb	Roadside	1
18	*Juglans regia* L.	Juglandaceae	Cryptogenic	Western Asia	P scap	Temporary water body/Roadside	5
19	*Kalanchoë delagoënsis* Eckl. & Zeyh.	Crassulaceae	Neophyte	Southern Africa	Ch succ	Roadside	1
20	*Luffa aegyptiaca* Mill.	Cucurbitaceae	Neophyte	Southern Africa	T scap	Temporary water body	1
21	*Narcissus ‘Cotinga’*	Amaryllidaceae	Neophyte	Culton	G bulb	Crop/Shrubland	2
22	*Narcissus ‘Erlicheer’*	Amaryllidaceae	Neophyte	Culton	G bulb	Roadside	1
23	*Nothoscordum gracile* (Aiton) Stearn	Amaryllidaceae	Neophyte	Southern America	G bulb	Roadside/Sidewalk	36
24	*Oxalis stricta* L.	Oxalidaceae	Neophyte	Northern America	H scap	Sidewalk	3
25	*Passiflora caerulea* L.	Passifloraceae	Neophyte	Southern America	P lian	Roadside	1
26	*Portulaca grandiflora* Hook.	Portulacaceae	Neophyte	Southern America	T scap	Roadside/Sidewalk	2
27	*Prunus armeniaca* L.	Rosaceae	Archeophyte	Culton	P scap	Crop	1
28	*Prunus dulcis* (Mill.) D.A. Webb	Rosaceae	Archeophyte	Feral	P scap	Outcropping rocks/Roadside/Shrubland	7
29	*Salpichroa origanifolia* (Lam.) Baill.	Solanaceae	Neophyte	Southern America	Ch frut	Roadside	10
30	*Sesbania punicea* (Cav.) Benth.	Fabaceae	Neophyte	Southern America	P scap	Temporary water body	1
31	*Solanum tuberosum* L.	Solanaceae	Neophyte	Culton	T scap	Temporary water body	1
32	*Tecoma stans* (L.) Juss. ex Kunth	Bignoniaceae	Neophyte	Northern America	P lian	Sidewalk	1
33	*Tradescantia sillamontana* Matuda	Commelinaceae	Neophyte	Southern America	G rhiz	Uncultivated field	1
34	*Washingtonia filifera* (Linden ex André) H. Wendl. ex de Bary	Arecaceae	Neophyte	Northern America	P scap	Sidewalk	1
						Total	**118**

Note. Plant life forms (according to the Raunkiaer system) Ch frut, Frutescent chamaephyte; Ch succ, Succulent chamaephyte; G bulb, Bulbous geophyte; G rhiz, Rhizome geophyte; H caesp, Cespitose hemicryptophyte; H scap, Scapose hemicryptophyte; NP, Nanophanerophyte; P lian, Climbing phanerophyte; P scap, Scapose phanerophyte; T scap, Scapose therophyte.

**Table 3:** Alien taxa found in two sites of the Natura 2000 network in Calabria (Southern Italy, Europe).

ID	Taxon	SAC/SPA	SAC/SPA Code	SAC/SPA Name
5	*Beta vulgaris* L. subsp. *vulgaris*	SAC	IT9350145	Fiumara Amendolea (incluso Roghudi, Chorio e Rota Greco)
5	*Beta vulgaris* L. subsp. *vulgaris*	SPA	IT9350300	Costa Viola
10	*Cedrus atlantica* (Endl.) G.Manetti ex Carrière	SPA	IT9350300	Costa Viola
15	*Fagopyrum esculentum* Moench	SPA	IT9350300	Costa Viola
16	*Freesia alba* (G.L. Mey.) Gumbl.	SPA	IT9350300	Costa Viola
18	*Juglans regia* L.	SPA	IT9350300	Costa Viola
22	*Nothoscordum gracile* (Aiton) Stearn	SPA	IT9350300	Costa Viola

Notes. SAC: Special Area of Conservation [60]; SPA: Special Protection Area [61].

**Figure A2:**
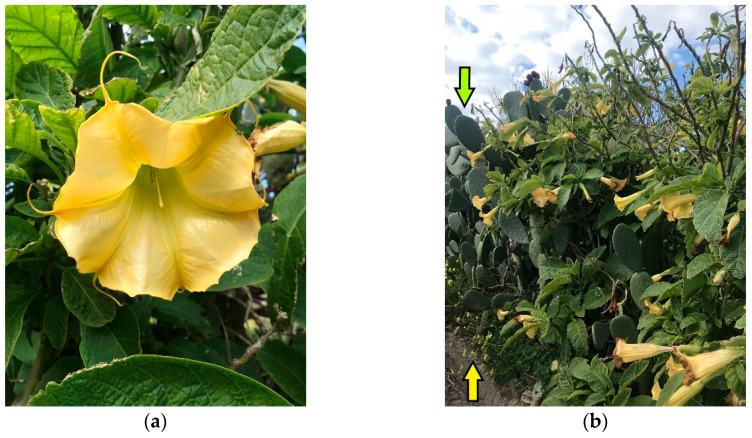
*Brugmansia aurea* Lagerh. found in a roadside in Brancaleone (Reggio Calabria province): (**a**) flower; (**b**) several individuals of *B. aurea* mixed with *Oxalis pes-caprae* L. (yellow arrow) and *Opuntia ficus-indica* (L.) Mill. (green arrow) (Photographs VLA Laface).

**Figure A3:**
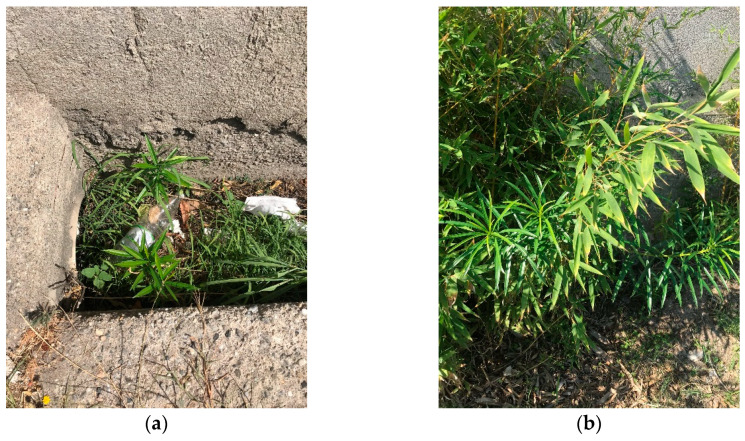
*Cascabela thevetia* (L.) Lippold found (**a**) in a water drainage channel in Melito di Porto Salvo (Reggio Calabria province) and (**b**) mixed with *Phyllostachys aurea* Carrière ex Rivière & C. Rivière in Reggio Calabria (Reggio Calabria province) (Photographs VLA Laface).

**Figure A4:**
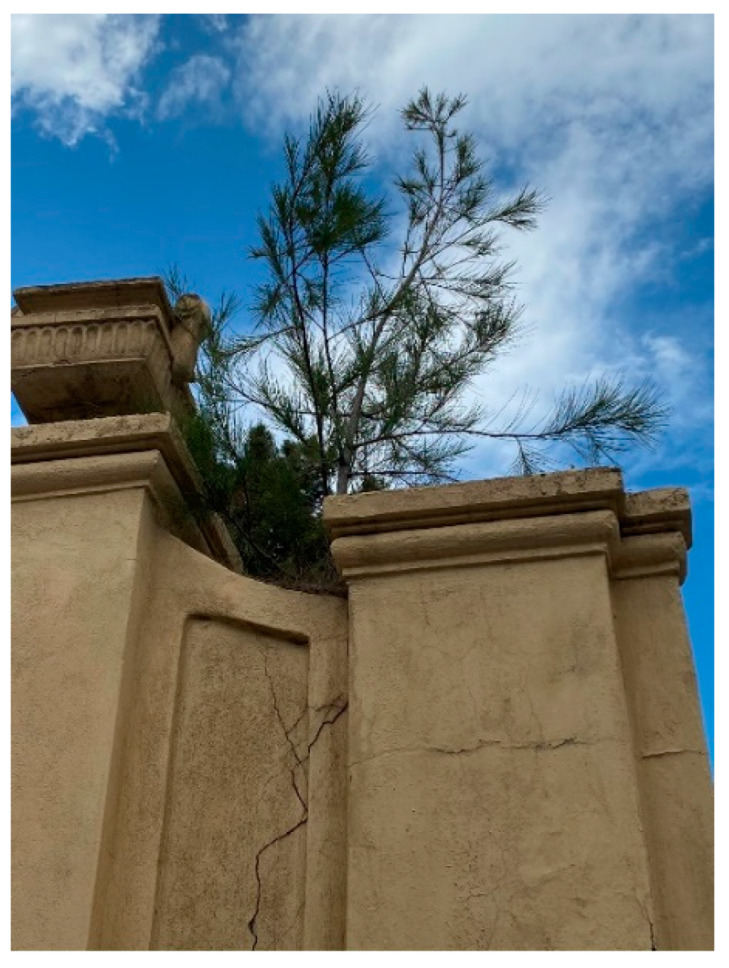
Juvenile of *Casuarina equisetifolia* L. on a wall in the city of Reggio Calabria.

**Figure A5:**
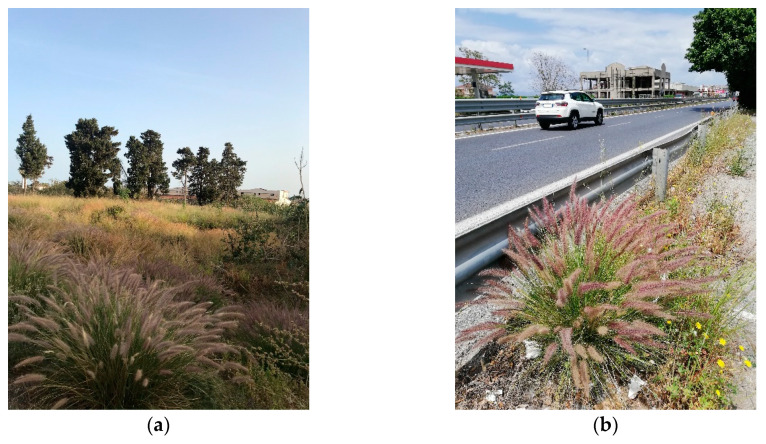
*Cenchrus setaceus* (Forssk.) Morrone widespread both (**a**) in a natural field in Saline Joniche (Reggio Calabria province) (Photograph VLA Laface) and (**b**) along a communication route in Reggio Calabria (Reggio Calabria province) (Photograph CM Musarella).

**Figure A6:**
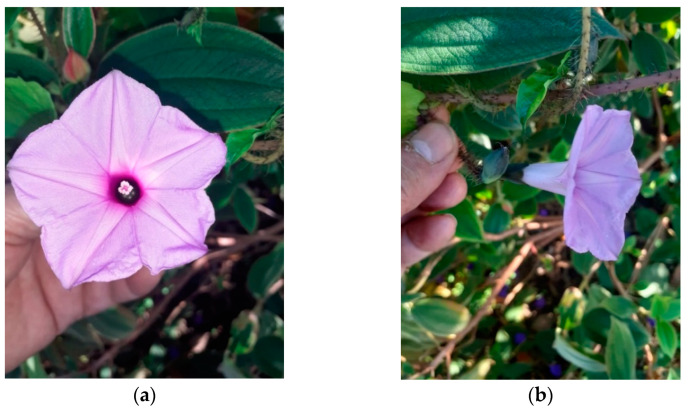

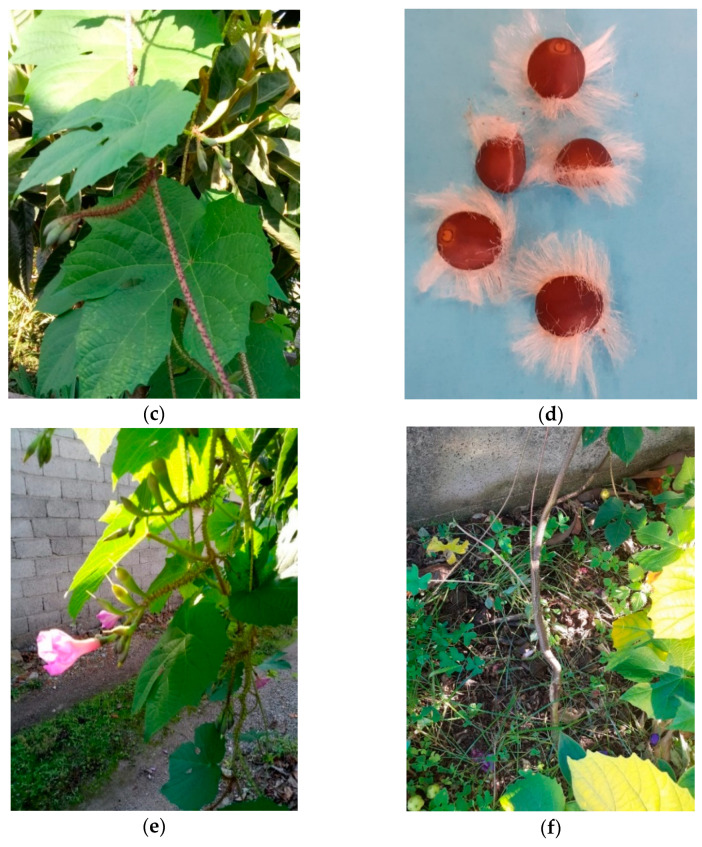
*Ipomoea setosa* Ker-Gawl. subsp. *pavonii* (Hallier f.) J.R.I. Wood & Scotland found for the first time in Europe in Rizziconi (Reggio Calabria province). (**a**) Flower in front view; (**b**) flower in side view; (**c**) stem and leaves; (**d**) hairy seeds; (**e**) location of the new record; (**f**) particular of the low part of the plant born along a rural way (Photographs S Cannavò).

**Figure A7:**
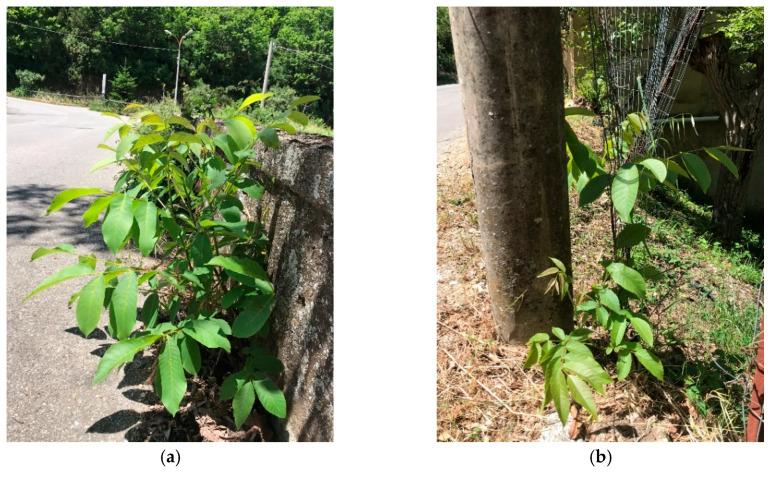
Juveniles of *Juglans regia* L. recorded along the roadside in Reggio Calabria province. (**a**) in Solano; (**b**) in Melia di Scilla (Photographs VLA Laface).

**Figure A8:**
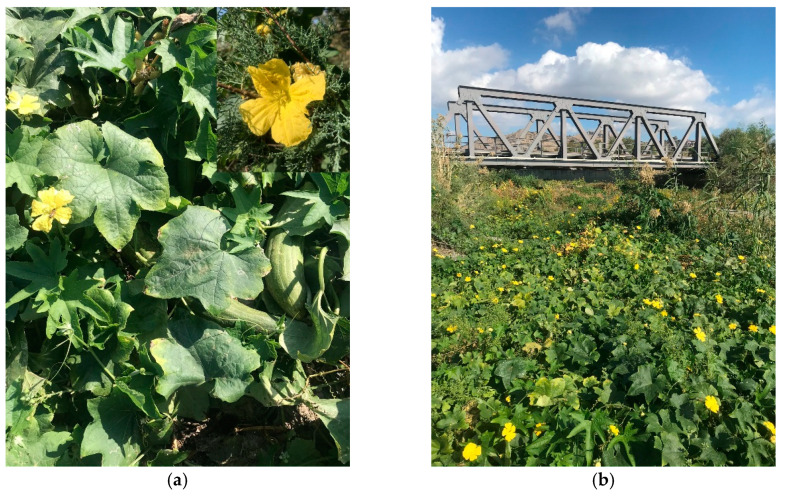
*Luffa aegyptiaca* Mill. in Fiumara Acrifa (Reggio Calabria province). (**a**) Immature fruits, leaves, and flowers; (**b**) several plants along the riverbed of the fiumara (Photographs VLA Laface).

**Figure A9:**
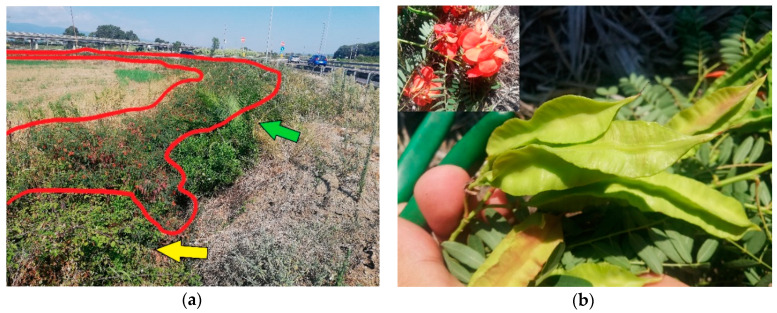
*Sesbania punicea* (Cav.) Benth. in a water drainage channel near the airport, between the way and a cultivated field in Lamezia Terme (Catanzaro province). (**a**) Bordered in red, part of the drainage channel occupied by *S. punicea*, together with *Rubus ulmifolius* Schott (yellow arrow) and an individual of *Phoenix canariensis* H. Wildpret (green arrow); (**b**) Fruits and, in the box above on the left, inflorescences of *S. punicea* of the new location of Lamezia Terme (Photographs CM Musarella).

**Figure A10:**
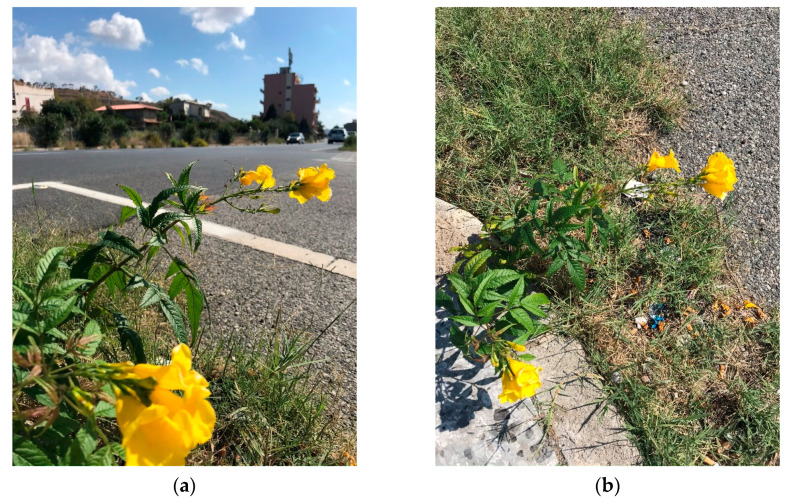
The individual of *Tecoma stans* (L.) Juss. ex Kunth collected in Bocale (Reggio Calabria province) along the sidewalk of S.S. Jonica 106. (**a**) *T. stans* and, in the background, the S.S. Jonica 106; (**b**) *T. stans* grown between the sidewalk and the road (Photographs VLA Laface).

**Figure A11:**
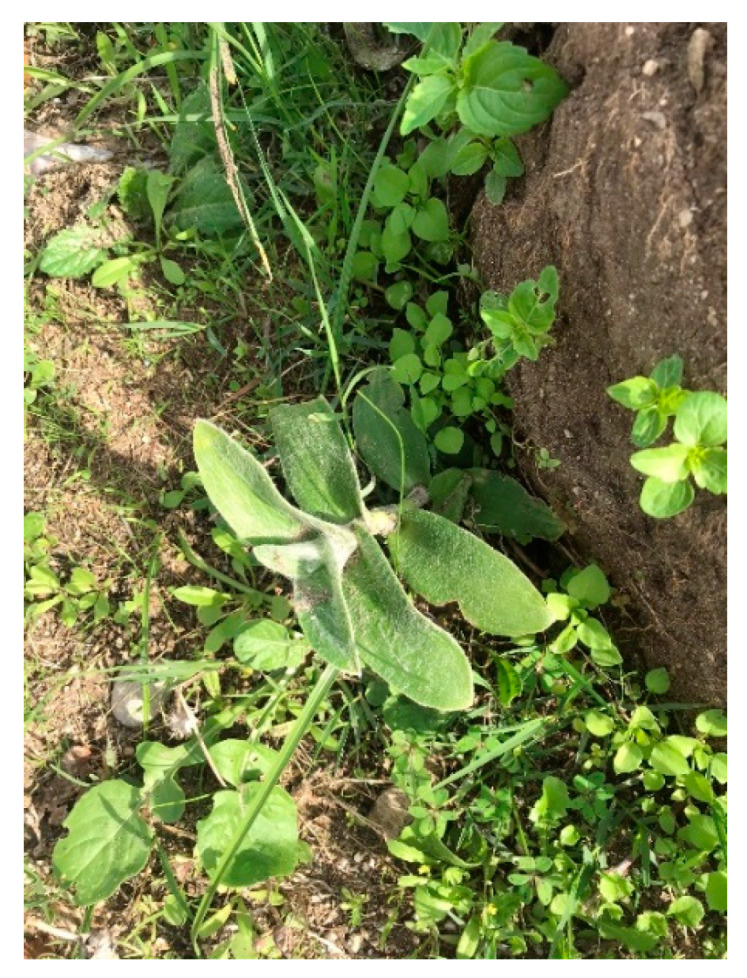
Juvenile of *Tradescantia sillamontana* Matuda found in a crevice in Contrada Monte Callea (Reggio Calabria province) (Photograph VLA Laface).

## Supplementary Materials

The following are available online at https://www.mdpi.com/2223-7747/9/9/1181/s1, Table S1: Details of all 118 new records.

## Appendix A

(1) ***Allium cepa* L.** [≡*Allium ascalonicum* auct., non L., *Allium esculentum* Salisb.]
  Amaryllidaceae Archeophyte Culton Bulbous geophyte
  First record for Calabria (casual)
  *Observatum*: 6 June 2019, Strada Comunale Amendolara (Reggio Calabria province), wheat field, 37.978678°N–15.818553°E, 348 m a.s.l., *obs. et det.* V.L.A. Laface.
  Note. Some flowering plants have been observed in a wheat field, which probably escaped cultivation from the vegetable gardens nearby. Casual alien in Abruzzo, Basilicata, Campania, Emilia-Romagna, Friuli Venezia Giulia, Lazio, Lombardia, Marche, Molise, Piemonte, Sardegna, Toscana, Umbria and Veneto, according to Galasso et al. [1].
(2) ***Araujia sericifera*****Brot.** [≡*Araujia albens* (Mart.) G. Don, *Araujia hortorum* E. Fourn.]
  Apocynaceae Neophyte Southern America Climbing phanerophyte
  First geolocalized reports in Calabria (casual)
  *Specimina*: 4 July 2019, Catona (Reggio Calabria province), roadside climbed to a wire mesh, 38.174222°N–15.649700°E, 25 m a.s.l., *leg. et det.* V.L.A. Laface (REGGIO); 29 July 2019, Località Zonzoloso, Borgata Musa (Reggio Calabria province), roadside climbed to a wire mesh, 37.933149°N–15.753858°E, 57 m a.s.l., *leg. et det.* V.L.A. Laface (REGGIO).
  Note. *A. sericifera* is present in Italy as naturalized in Abruzzo and Sicilia, as casual in Lazio, Lombardia, Marche, Molise, Puglia, Sardegna, and Toscana, and as invasive in Campania [1]. In Calabria, this species was indicated as “recently not confirmed” by Celesti-Grapow et al. [87] and Galasso et al. [1], whereas was recently reported by Musarella et al. [44] for the same locality of Catona as casual, but in an unspecified uncultivated field. Recently, Lazzaro et al. [62] proposed this species for its inclusion in a national list of invasive species according to EU Regulation 1143/2014 [88].
(3) ***Asparagus setaceus* (Kunth) Jessop** [≡*Asparagopsis setacea* Kunth]
  Asparagaceae Neophyte Southern Africa Climbing phanerophyte
  First record for Calabria (casual)
  *Specimina*: 2 March 2020, Località Bolano, tra Catona e Villa San Giovanni (Reggio Calabria province), citrus grove abandoned for many years, 38.203382°N–15.635614°E, 7 m a.s.l., *leg. et det.* V.L.A. Laface (REGGIO); 4 March 2020, Catona (Via Feudo) (Reggio Calabria province), climbed to a wire mesh, 38.185416°N–15.646368°E, 16 m a.s.l., *leg. et det.* V.L.A. Laface (REGGIO); 3 March 2020, Gallico Marina (Reggio Calabria province), abandoned citrus grove, 38.164632°N–15.649939°E, 9 m a.s.l., *leg. et det.* V.L.A. Laface (REGGIO); 11 May 2020, Catona (Reggio Calabria province), climbed to a wire mesh, 38.188973°N–15.642679°E, 37 m a.s.l., *leg. et det.* V.L.A. Laface (REGGIO).
  *Observata*: 3 March 2020, Gallico Inferiore (Reggio Calabria province), uncultivated garden, 38.166028°N–15.653223°E, 16 m a.s.l., *obs. et det.* V.L.A. Laface.
  Note. Used as an ornamental plant, probably escaped from the surrounding gardens. In the above localities, it was found in an old citrus grove and in abandoned lands where it also climbs over the existing trees. It is distributed in different regions of the Italian Peninsula as naturalized in Campania and Sicilia, whereas it is casual in Lazio, Puglia, Sardegna, Trentino-Alto Adige, and Toscana [1].
(4) ***Bassia scoparia* (L.) Voss** [≡*Chenopodium scoparia* L.]
  Chenopodiaceae Neophyte Central Asia Scapose therophyte
  First record for Calabria (casual)
  *Specimen*: 21 October 2019, Via Torrente Carrò e Quattrone, Pellaro (Reggio Calabria province), roadside, 38.026944°N–15.656278°E, 16 m a.s.l., *leg. et det.* C.M. Musarella (REGGIO).
  Note. Widely used as an ornamental plant, particularly appreciated for the creation of low hedges, it is probably escaped from plants grown in pots or from nearby flowerbeds (observed by Musarella). *B. scoparia* is present throughout Italy as invasive, naturalized, or casual alien and not reported only for Molise and Basilicata [1].
(5) ***Beta vulgaris* L. subsp. *vulgaris*** [≡*Beta cicla* L.; *Beta esculenta* Salisb.; *Beta hortensis* Mill.; *Beta rapa* Dumort.]
  Chenopodiaceae Archeophyte Culton Scapose hemicryptophyte
  First record for Calabria (naturalized)
  *Specimen*: 28 February 2020, Ortì Inferiore (Reggio Calabria province), orchard, 38.147958°N–15.709523°E, 604 m a.s.l., *leg. et det.* V.L.A. Laface (REGGIO).
  *Observata*: 26 January 2020, Via Provinciale Sant’Elia-Montebello Jonico (Reggio Calabria province), roadside, 37.954042°N–15.754001°E, 230 m a.s.l., *obs. et det.* V.L.A. Laface; 9 February 2020, C.da Pellegrino, Fiumara Acrifa (Reggio Calabria province), bergamot grove, 37.926021°N–15.839961°E, 17 m a.s.l., *obs. et det.* V.L.A. Laface; 9 February 2020, Bova Marina, vicino la Fiumara Amendolea (Reggio Calabria province), bergamot grove, 37.933048°N–15.893546°E, 17 m a.s.l., *obs. et det.* V.L.A. Laface; 19 January 2020, San Fantino (Reggio Calabria province), uncultivated field, 38.001735°N–15.817078°E, 329 m a.s.l., *obs. et det.* V.L.A. Laface; 22 January 2020, San Pantaleone Località Varada (Reggio Calabria province), uncultivated field, 37.989366°N–15.831174°E, 544 m a.s.l., *obs. et det.* V.L.A. Laface; 10 June 2019, Solano Superiore, Bagnara (Reggio Calabria province), roadside, 38.248756°N–15.794914°E, 653 m a.s.l., *obs. et det.* V.L.A. Laface.
  Note. *B. vulgaris* subsp. *vulgaris* is cultivated throughout Italy for food purposes. It grows abundantly in uncultivated and cultivated fields, in abandoned lands and on the edges of country roads. The subspecies is considered as a casual alien throughout Italy [1]. Several records above reported are known by Laface since about twenty years for their own domestic food uses.
(6) ***Bidens formosa* (Bonato) Sch. Bip.** [≡*Coreopsis formosa* Bonato; *Cosmos bipinnatus* Cav., non *Bidens bipinnata* L.; *Cosmos bipinnatus* Cav.]
  Asteraceae Neophyte Northern and Central America Scapose therophyte
  First record for Calabria (casual)
  *Specimina*: 29 February 2020 Località Cazzeria, Catona (Reggio Calabria province), roadside, 38.191548°N–15.646137°E, 25 m a.s.l., *leg. et det.* V.L.A. Laface (REGGIO); 29 February 2020, Località Cazzeria, Catona (nei pressi del ponte dell’autostrada A3) (Reggio Calabria province), roadside, 38.192187°N–15.647553°E, 28 m a.s.l., *leg. et det.* V.L.A. Laface (REGGIO).
  Note. *B. formosa* is reported as a casual alien in regions of northern and central Italy [1].
(7) ***Brugmansia aurea* Lagerh.** [≡*Brugmansia pittieri* (Saff.) Moldenke; *Brugmansia affinis* (Soff.) Moldenke]
  Solanaceae Neophyte Ecuador Nanophanerophyte
  First record for Italy (casual)
  *Specimen*: 11 January 2020, S.S. Jonica 106, Galati, Brancaleone (Reggio Calabria province), roadside, 37.932976°N–16.069568°E, 8 m a.s.l., *leg. et det.* V.L.A. Laface (REGGIO).
  Note. Cultivated for ornamental purposes, very fragrant trumpet-shaped flowers, this plant is very attractive to bees, butterflies, and birds. Two plants were recorded along the road, mixed with *Opuntia ficus-indica* (L.) Mill. and *Oxalis pes-caprae* L. (Appendix B, Figure A2). In Europe, *B. aurea* was recorded only in Spain [89], therefore, this is the second report for Europe and the first for Italy.
(8) ***Cascabela thevetia* (L.) Lippold** [≡*Cascabela peruviana* (Pers.) Raf.; *Thevetia peruviana* (Pers.) K. Schum.]
  Apocynaceae Neophyte Central and Southern America Frutescent chamaephyte
  First record for Europe (casual)
  *Specimina*: 5 October 2019, Melito di Porto Salvo (Reggio Calabria province), water drainage channel, 37.920423°N–15.802782°E, 9 m a.s.l., *leg. et det.* V.L.A. Laface (REGGIO); 19 October 2019, Fiumara Acrifa (Reggio Calabria province), near a fiumara, 37.920494°N-15.839534°E, 9 m a.s.l., *leg. et det.* V.L.A. Laface (REGGIO).
  Note. *C. thevetia* is an evergreen shrub native to tropical America (Mexico, Central America (Guatemala, Belize, El Salvador, Honduras, Nicaragua, Costa Rica, and Panama), South America (Colombia, Venezuela, British Guiana, Brazil, Ecuador, Peru, and Bolivia) and cultivated in the Antilles (Bahamas, Cuba, Haiti, Jamaica, Puerto Rico, and Dominican Republic), but it is widespread in the tropics around the world [90]. It is commonly cultivated as an ornamental plant and its flowering occurs throughout the year. Numerous seedlings have been found, born from seeds, in a water drainage channel and some adult plants along a wall, on the side of a typical little river (“fiumara”), mixed with *Phyllostcahys aurea* Carrière ex Rivière and C. Rivière (Appendix B, Figure A3). In Europe, it is reported as doubtful for Cyprus [91] and a preserved specimen is recorded for Portugal, but without a certain locality [92]. However, according to [83,84], *C. thevetia* is not given as present in Cyprus. Therefore, our finding represents the first European record.
(9) ***Casuarina equisetifolia* L.**
  Casuarinaceae Neophyte Australia Scapose phanerophyte
  First record for Calabria (casual)
  *Observata*: 29 December 2019, Reggio Calabria (incrocio Via Aschenez-Via Willermin) (Reggio Calabria province), wall, 38.115494°N–15.654883°E, 35 m a.s.l., *obs. et det.* V.L.A. Laface; 18 January 2019, Straci, Marina di San Lorenzo (Reggio Calabria province), wall of a traditional water collection tank (“gebbia”), 37.923677°N–15.844383°E, 19 m a.s.l., *obs. et det.* V.L.A. Laface.
  Note. The plants observed were certainly generated by the seeds of some trees nearby planted, accidentally blown into the cracks in the walls (Appendix B, Figure A4). According to Galasso et al. [1], in Italy, this species is a casual alien in Lazio, Sicilia, Campania, and naturalized in Puglia and Toscana.
(10) ***Cedrus atlantica* (Endl.) G. Manetti ex Carrière** [≡*Pinus atlantica* Endl.]
  Pinaceae Neophyte Northern Africa Scapose phanerophyte
  First record for Calabria (casual)
  *Specimen*: 9 May 2020, Piani di Reggio, Reggio Calabria (Reggio Calabria province), slope, 38.145513°N–15.803511°E, 1163m a.s.l., *leg. et det.* V.L.A. Laface.
  *Observata*: 6 May 2019, S.S. 184, Santo Stefano in Aspromonte (Reggio Calabria province), roadside and beech wood, 38.170283°N–15.828692°E, 1211m a.s.l., *obs. et det.* V.L.A. Laface.
  Note. According to Galasso et al. [1], in Italy, this species is a casual alien only in Sicilia and Basilicata.
(11) ***Cenchrus setaceus* (Forssk.) Morrone** [≡*Phalaris setacea* Forssk.; *Pennisetum setaceum* (Forssk.) Chiov.]
  Poaceae Neophyte Northern and Eastern Africa and Arabian Peninsula Cespitose hemicryptophyte
  Change of status for Calabria: from naturalized to invasive (invasive)
  *Specimina*: 15 February 2020, Occhio di Pellaro (Reggio Calabria province), roadside, 38.043684°N–15.656055°E, 6 m a.s.l., *leg. et det.* C.M. Musarella (REGGIO); 8 May 2020, S.S. 106 (Reggio Calabria province), roadside, 38.064363°N–15.662721°E, 40 m a.s.l., *leg. et det.* C.M. Musarella (REGGIO); 17 May 2020, Saline Joniche (Reggio Calabria province), roadside, 37.944089°N–15.703504°E, 11 m a.s.l., *leg. et det.* V.L.A. Laface (REGGIO); 2 June 2020, C.da Venturi, Bova (Reggio Calabria province), roadside and uncultivated field, 37.937291°N–15.942257°E, 144 m a.s.l., *leg. et det.* V.L.A. Laface (REGGIO); 02-06-2020, Località Scilindermeno, Bova (Reggio Calabria province), roadside and wall, 37.953626°N–15.947259°E, 230 m a.s.l., *leg. et det.* V.L.A. Laface (REGGIO); 02-06-2020, Coddurena, Bova (Reggio Calabria province), roadside, 37.968476°N–15.947824°E, 398 m a.s.l., *leg. et det.* V.L.A. Laface (REGGIO).
  Note. *C. setaceus* is one of the “black” species present in the “Union list” of the European Union [86]. In Italy, this species is a casual alien in Calabria, Lazio, and Toscana, naturalized in Puglia, and invasive in Sardegna and Sicilia [1]. Subsequently, Musarella et al. [44] reported it as naturalized in Calabria. The specimens collected (S.S. 106 Occhio di Pellaro and Saline Joniche) along some city streets (Appendix B, Figure A5) probably come from some nearby villas or other spontaneous individuals geographically close. The new records reported here previously had not been observed by the authors, which means that they were born less than a year after previous reports by Musarella et al. [44].
  Reported for the first time in Calabria in 2007 [64], the species shows (as in other Italian regions) a strongly invasive character. *C. setaceus* was initially reported for this region by Celesti-Grapow et al. [87] and then confirmed by Musarella et al. [49] as casual. Recently, it was recorded as naturalized, according to Musarella et al. [44], but this species continues its uncontrollable expansion in Calabria and can therefore be considered as invasive.
(12) ***Chlorophytum comosum* (Thunb.) Jacques** [≡*Anthericum comosum* Thunb.]
  Asparagaceae Neophyte Southern Africa Scapose hemicryptophyte
  First record for Calabria (casual)
  *Specimina*: 22 February 2020, Località Santa Maria, Ortì Inferiore (Reggio Calabria province), water drainage channel, 38.147425°N–15.711690°E, 608 m a.s.l., *leg. et det.* V.L.A. Laface (REGGIO); 22 February 2020, Località Santa Maria, Ortì Inferiore (Reggio Calabria province), rushes in an abandoned field, 38.147170°N–15.711247°E, 598 m a.s.l., *leg. et det.* V.L.A. Laface.
  Note. Several plants have been observed, most likely escaped from some pots nearby. The species has always been observed near wetlands. In Italy, this species is recorded as a casual alien in Abruzzo, Lombardia and Sardegna, and as naturalized in Campania [1].
(13) ***Cucurbita maxima* Duchesne subsp. *maxima***
  Cucurbitaceae Neophyte Culton Scapose therophyte
  First record for Calabria (casual)
  *Observata*: 23 July 2019, Sambatello (Reggio Calabria province), roadside, 38.179022°N–15.694885°E, 297 m a.s.l., *obs. et det.* V.L.A. Laface; 06.06.2019, Ortì Inferiore (Reggio Calabria province), water drainage channel, 38.147552°N–15.710772°E, 604 m a.s.l., *obs. et det.* V.L.A. Laface.
  Note. *C. maxima* is locally cultivated for food purposes. Some flowering plants have been observed near water sources, likely to have escaped cultivation from nearby vegetable gardens. According to Galasso et al. [1], this subspecies is considered as a casual alien in northern Italy, in some regions of central Italy, and on the islands.
(14) ***Dolichandra unguis-cati* (L.) L.G. Lohmann** [≡*Bignonia unguis-cati* L.; *Macfadyena unguis-cati* (L.) A.H. Gentry]
  Bignoniaceae Neophyte Southern America Climbing phanerophyte
  First record for Calabria (Casual)
  *Specimina*: 4 July 2019, Gallico (Reggio Calabria province), ruins, 38.146874°N–15.660094°E, 28 m a.s.l., *leg. et det.* V.L.A. Laface (REGGIO); 26 January 2020, Bova Marina (Reggio Calabria province), climbed to a wire mesh and ruins, 37.930419°N–15.915407°E, 9 m a.s.l., *leg. et det.* V.L.A. Laface (REGGIO); 26 January 2020, Località Vena, Bova Marina (Reggio Calabria province), climbing among the bricks of a bridge and in a water drainage channel, 37.929289°N–15.908896°E, 9 m a.s.l., *leg. et det.* V.L.A. Laface (REGGIO).
  Note. *D. unguis-cati* is present in Italy as a casual alien only in Campania, Lazio, Liguria, Sicilia, and Toscana [1].
(15) ***Fagopyrum esculentum* Moench** [≡*Fagopyrum sagittatum* Gilib.; *Fagopyrum vulgare* Hill; *Polygonum fagopyrum* L.]
  Polygonaceae Neophyte Asia Scapose therophyte
  First record for Calabria (casual)
  *Specimen*: 5 May 2019, Lucia, Laganadi (Reggio Calabria province), roadside, 38.206843°N–15.756740°E, 388 m a.s.l., *leg. et det.* V.L.A. Laface (REGGIO).
  Note. Few plants were recorded along a road, mixed with *Amaranthus* sp. and *Setaria* sp., probably escaped from the cultivation of nearby gardens or vegetable gardens. In Italy, it is present in many regions as a casual alien (Abruzzo, Campania, Emilia-Romagna, Friuli Venezia Giulia, Lazio, Lombardia, Piemonte, Puglia, Trentino-Alto Adige, Umbria, and Veneto), whereas in Toscana it is naturalized [1].
(16) ***Freesia alba* (G.L. Mey.) Gumbl.** [≡*Freesia refracta* var. *alba* G.L. Mey.; *Freesia leichtlinii* subsp. *alba* (G.L. Mey.) J.C. Manning & Goldblatt]
  Iridaceae Neophyte Southern Africa Bulbous geophyte
  First record for Calabria (casual)
  *Specimina*: 2 March 2020, Località San Cono, Rosalì (Reggio Calabria province), drywall limiting a “fiumara”, 38.202119°N–15.671522°E, 94 m a.s.l., *leg. et det.* V.L.A. Laface (REGGIO); 4 March 2020, Ortì Superiore (Reggio Calabria province), roadside, 38.151084°N–15.731452°E, 668 m a.s.l., *leg. et det.* V.L.A. Laface (REGGIO).
  *Observata*: 9 February 2020, Località Trabocchetto, Reggio Calabria (Reggio Calabria province), in a gutter, 38.108561°N–15.652622°E, 82 m a.s.l., *obs. et det.* V.L.A. Laface.
  Note. A South African species [80], present in Italy as casual alien in Abruzzo, Basilicata, Lazio, Liguria, Molise, Puglia, Sardegna, Sicilia, Toscana and as naturalized in Campania [1].
(17) ***Ipomoea setosa* Ker-Gawl. subsp. *pavonii* (Hallier f.) J.R.I. Wood & Scotland** [≡*Calonyction pavonii* Hallier f.; *Merremia pavonii* (Hallier f.) D.F. Austin & Staples; *Ipomoea setosa* var. *pavonii* (Hallier f.) House]
  Convolvulaceae Neophyte Southern America Bulbous geophyte
  First record for Europe (casual)
  *Specimen*: 18 October2019, C.da La Monaca, Rizziconi (Reggio Calabria province), roadside, 38.418530°N–15.967880°E, 81 m a.s.l., *leg*. S. Cannavò, *det*. S. Cannavò, V.L.A. Laface, C.M. Musarella *et* G. Spampinato (REGGIO).
  Note. Recently, Wood et al. [81] published a monograph on *Ipomoea* genus in the New World. In it, the authors describe 425 taxa of *Ipomoea* and provide an identification key. According to these authors, the collected specimen from Rizziconi belong to *I. setosa* subsp. *pavonii*, a new recognized subspecies (*comb. et stat. nov.*). This subspecies is native to South America and it has fleshy trichomes on peduncles and pedicels, and these last are strongly swollen below the calyx. Its flowers have a short corolla of 5–6.5 cm [81]. Some details of the plants collected in Calabria are presented in Figure A6 of Appendix B. Until this record, *Ipomoea setosa* subsp. *pavonii* had not been recorded in Europe. Thus, our finding represents the first European record, according to Euro+Med PlantBase [83] and DAISIE (Inventory of alien invasive species in Europe) [sub *Merremia pavonii* (Hallier f.) D.F. Austin & Staples] [91].
(18) ***Juglans regia* L.**
  Juglandaceae Cryptogenic Western Asia Scapose phanerophyte
  First record for Calabria (casual)
  *Specimina*: 16 February 2020, Cicala (Catanzaro province), escarpment, 39.024162°N–16.503332°E, 885 m a.s.l., *leg.* D. Grande, *det.* C.M. Musarella (REGGIO); 14 March 2020, Fiumara Catona, San Roberto (Reggio Calabria province), riverbed, 38.208153°N–15.754430°E, 374 m a.s.l., *leg. et det.* V.L.A. Laface (REGGIO); 09.05.2020, San Roberto (Reggio Calabria province), roadside, 38.208000°N–15.749657°E, 349 m a.s.l., *leg. et det.* V.L.A. Laface (REGGIO); 25 May 2020, Villaggio del Pino, Melia di Scilla, (Reggio Calabria province), roadside, 38.237782°N–15.737439°E, 600 m a.s.l., *leg. et det.* V.L.A. Laface (REGGIO); 25 May 2020, Solano (Reggio Calabria province), roadside, 38.251673°N–15.797604°E, 601 m a.s.l., *leg. et det.* V.L.A. Laface (REGGIO).
  Note: Some adult plants have been observed in both localities of Cicala and San Roberto, whereas juveniles were found in Solano and Melia di Scilla (Appendix B, Figure A7). In Italy, based on Galasso et al. [1], *J. regia* is present as naturalized in almost all regions of the Italian Peninsula or as cryptogenic in Sardegna, Lombardia, and Veneto, whereas it is absent only in Puglia. In Calabria, its use is known not only for their fruits as food but also for their leaves as an emollient for excessive feet perspiration [33,35] or to treat warts [34].
(19) ***Kalanchoë delagoënsis* Eckl. & Zeyh.** [≡*Bryophyllum delagoense* (Eckl. & Zeyh.) Druce; *Kalanchoë delagoensis* Eckl. & Zeyh.]
  Crassulaceae Neophyte Southern Africa Succulent chamaephyte
  First record for Calabria (casual)
  *Specimen*: 15 February 2020, Via Sottolume, Pellaro (Reggio Calabria province), roadside, 38.021766°N–15.648545°E, 15 m a.s.l., *leg. et det.* C.M. Musarella (REGGIO).
  Note. Some plants have been observed on roadsides, probably originating from some nearby pots where the plant is grown. According to Galasso et al. [1], this species is present as a casual alien in Sicilia, Sardegna, Lazio, and Puglia.
(20) ***Luffa aegyptiaca* Mill.** [≡*Luffa cylindrica* M. Roem., non*-Momordica cylindrica* L.; *Momordica luffa* L.]
  Cucurbitaceae Neophyte Southern Africa Scapose therophyte
  First record for Calabria and Italian Peninsula (casual)
  *Specimen*: 19 October 2019, Fiumara Acrifa (Reggio Calabria province), riverbed, 37.920592°N–15.839740°E, 9 m a.s.l., *leg. et det.* V.L.A. Laface (REGGIO).
  Note. Several plants have been found occupying a large part of the river (“fiumara”) and almost completely covered the vegetation below, characterized mainly by shrubs of *Tamarix africana* Poir (Appendix B, Figure A8). Based on Galasso et al. [1], *L. aegyptiaca* is present exclusively in Sicilia as a casual alien, therefore, this is the first report for Calabria and the Italian Peninsula.
(21) ***Narcissus ‘Cotinga’***
  Amaryllidaceae Neophyte Culton Bulbous geophyte
  First record for Italy (naturalized)
  *Specimina*: 27 April 2019, Piani di Reggio (Reggio Calabria province), grazing-meadow covered with broom, 38.136339°N–15.823482°E, 1313 m a.s.l., *leg. et det.* V.L.A. Laface (REGGIO); 14 March 2020, Podargoni (Reggio Calabria province), abandoned walnut grove, 38.164593°N–15.780524°E, 543 m a.s.l., *leg.* V.L.A. Laface, *det.* C.M. Musarella (REGGIO).
(22) ***Narcissus ‘Erlicheer’***
  Amaryllidaceae Neophyte Culton Bulbous geophyte
  First record for Italy (casual)
  *Specimen*: 24 January 2020, Via Torrente Carrò e Quattrone, Pellaro (Reggio Calabria province), roadside, 38.027402°N–15.657049°E, 18 m a.s.l. *leg. et det.* C.M. Musarella (REGGIO).
(23) ***Nothoscordum gracile* (Aiton) Stearn** [≡*Allium gracile* Aiton; *Nothoscordum inodorum* (Aiton) G. Nicholson; *Nothoscordum borbonicum sensu Auct. Fl. Ital.*, non Kunth]
  Amaryllidaceae Neophyte Southern America Bulbous geophyte
  Change of status for Calabria: from casual to invasive (invasive)
  *Specimina*: 5 March 2020, Archi Carmine (Reggio Calabria province), roadside, 38.155941°N–15.662968°E, 34 m a.s.l., *leg. et det.* V.L.A. Laface (REGGIO); 9 May 2020, Catona (Reggio Calabria province), roadside, 38.187991°N–15.640659°E, 9 m a.s.l., *leg. et det.* V.L.A. Laface (REGGIO); 9 May 2020, Catona (Reggio Calabria province), roadside, 38.186554°N–15.639327°E, 5 m a.s.l., *leg. et det.* V.L.A. Laface (REGGIO); 09 May 2020, Catona (Reggio Calabria province), roadside, 38.185641°N–15.638498°E, 3 m a.s.l., *leg. et det.* V.L.A. Laface (REGGIO); 9 May 2020, Catona (Reggio Calabria province), roadside, 38.182539°N–15.640183°E, 1 m a.s.l., *leg. et det.* V.L.A. Laface (REGGIO); 9 May 2020, Catona (Reggio Calabria province), roadside, 38.173234°N–15.640023°E, 1 m a.s.l., *leg. et det.* V.L.A. Laface (REGGIO); 10 May 2020, Località San Francesco, Catona (Reggio Calabria province), roadside, 38.181355°N–15.647343°E, 23 m a.s.l., *leg. et det.* V.L.A. Laface (REGGIO); 10 May 2020, Gallico Inferiore (Reggio Calabria province), roadside, 38.172231°N–15.651144°E, 34 m a.s.l., *leg. et det.* V.L.A. Laface (REGGIO); 10 May 2020, Gallico Inferiore (Reggio Calabria province), roadside, 38.172078°N–15.651199°E, 23 m a.s.l., *leg. et det.* V.L.A. Laface (REGGIO); 10 May 2020, Località S. Antonino, Gallico Inferiore (Reggio Calabria province), roadside, 38.169784°N–15.651662°E, 18 m a.s.l., *leg. et det.* V.L.A. Laface (REGGIO); 10 May 2020, Gallico Inferiore (Reggio Calabria province), roadside and wall, 38.167760°N–15.652999°E, 19 m a.s.l., *leg. et det.* V.L.A. Laface (REGGIO); 10-05-2020, Ponte Itria, Gallico Inferiore (Reggio Calabria province), roadside, 38.164272°N–15.654143°E, 15 m a.s.l., *leg. et det.* V.L.A. Laface (REGGIO); 10 May 2020, Gallico Inferiore (Reggio Calabria province), roadside, 38.158512°N–15.656363°E, 15 m a.s.l., *leg. et det.* V.L.A. Laface (REGGIO); 10 May 2020, Scaccioti, Archi (Reggio Calabria province), roadside, 38.156560°N–15.660512°E, 26 m a.s.l., *leg. et det.* V.L.A. Laface (REGGIO); 10 May 2020, Campo Calabro (Reggio Calabria province), roadside, 38.215613°N–15.658041°E,139 m a.s.l., *leg. et det.* V.L.A. Laface (REGGIO); 10 May 2020, Campo Calabro (Reggio Calabria province), roadside, 38.215233°N–15.656918°E, 142 m a.s.l., *leg. et det.* V.L.A. Laface (REGGIO); 10 May 2020, Concessa (Reggio Calabria province), roadside, 38.193435°N–15.641554°E, 30 m a.s.l., *leg. et det.* V.L.A. Laface (REGGIO); 10 May 2020, Concessa (Reggio Calabria province), roadside, 38.192726°N–15.641197°E, 33 m a.s.l., *leg. et det.* V.L.A. Laface (REGGIO); 10 May 2020, Concessa (Reggio Calabria province), roadside, 38.192295°N–15.640988°E, 24 m a.s.l., *leg. et det.* V.L.A. Laface (REGGIO); 10 May 2020, Concessa (Reggio Calabria province), roadside, 38.192072°N-15.640936°E, 22 m a.s.l., *leg. et det.* V.L.A. Laface (REGGIO); 10 May 2020, Villa San Giovanni (Reggio Calabria province), roadside, 38.215063°N–15.638823°E, 27 m a.s.l., *leg. et det.* V.L.A. Laface (REGGIO); 8 May 2020, Lazzaro (Reggio Calabria province), roadside, 37.974493°N–15.664018°E, 17 m a.s.l., *leg. et det.* C.M. Musarella (REGGIO); 08 May 2020, Lazzaro (Reggio Calabria province), roadside, 37.974547°N–15.664240°E, 18 m a.s.l., *leg. et det.* C.M. Musarella (REGGIO); 8 May 2020, Lazzaro (Reggio Calabria province), roadside, 37.974068°N–15.664895°E, 17 m a.s.l., *leg. et det.* C.M. Musarella (REGGIO); 8 May 2020, Lazzaro (Reggio Calabria province), roadside, 37.974218°N–15.665000°E, 18 m a.s.l., *leg. et det.* C.M. Musarella (REGGIO); 13 May 2019, Viale Umberto Zanotti Bianco, Villa San Giovanni (Reggio Calabria province), sidewalk, 38.214597°N–15.637022°E, 8 m a.s.l., *obs. et det.* V.L.A. Laface (REGGIO); 13 May 2020, Viale Umberto Zanotti Bianco, Villa San Giovanni (Reggio Calabria province), roadside, 38.213352°N–15.636946°E, 10 m a.s.l., *leg. et det.* V.L.A. Laface (REGGIO); 13 May 2020, Viale Umberto Zanotti Bianco, Villa San Giovanni (Reggio Calabria province), roadside, 38.212765°N–15.637123°E, 9 m a.s.l., *leg. et det.* V.L.A. Laface (REGGIO); 13 May 2020, Acciarello, Villa San Giovanni (Reggio Calabria province), roadside, 38.208752°N–15.635923°E, 9 m a.s.l., *leg. et det.* V.L.A. Laface (REGGIO); 13 May 2020, Bolano, tra Catona e Villa S. Giovanni (Reggio Calabria province), roadside, 38.205922°N–15.635899°E, 5 m a.s.l., *leg. et det.* V.L.A. Laface (REGGIO); 13 May 2020, Spontone, Catona (Reggio Calabria province), roadside, 38.192740°N–15.636915°E, 11 m a.s.l., *leg. et det.* V.L.A. Laface (REGGIO); 13 May 2020, Sambatello (Reggio Calabria province), roadside, 38.177942°N–15.690210°E, 270 m a.s.l., *leg. et det.* V.L.A. Laface (REGGIO).
  Note. *N. gracile* has been observed growing in the crevices of sidewalks, probably escaped from nearby pots or flower beds. In Italy, it is present as naturalized in Campania, Toscana, and Puglia and as a casual alien in Liguria, Piemonte, and Sardegna [1]. *N. gracile* was recently reported in Calabria by Rosati et al. [52] as casual in Lazzaro (Reggio Calabria province). In several locations reported here in *Specimina* (and in particular in Lazzaro), this species has spread everywhere, especially along the edges of the streets. Therefore, due to its rapid spread, we propose here the new status of invasive.
(24) ***Oxalis stricta* L.** [≡*Oxalis europaea* Jord.; *Oxalis fontana* Bunge; *Xanthoxalis stricta* (L.) Small; *Oxalis corniculate* var. stricta (L.) C.C. Huang & L.R. Xu; *Oxalis stricta* f. *lejeunei* Rouy, *Oxalis chinensis* Haw. ex G. Don; *Oxalis cymose* Small; *Oxalis lejeunei* (Rouy) A.W. Hill; *Xanthoxalis cymose* (Small) Small; *Xanthoxalis europaea* (Jord.) Moldenke; *Xanthoxalis fontana* (Bunge) Holub]
  Oxalidaceae Neophyte Northern America Scapose hemicryptophyte
  Confirmation for Calabria (casual)
  *Specimen*: 14 March 2020, Via della Fonderia, Catona (Reggio Calabria province), sidewalk in a roadside, 38.189965°N–15.644670°E, 17 m a.s.l., *leg. et det.* V.L.A. Laface (REGGIO).
  *Observata*: 4 March 2020, Occhio di Pellaro (Reggio Calabria province), sidewalk, 38.035157°N–15.657916°E, 7 m a.s.l., *obs. et det.* V.L.A. Laface; 28 March 2020, Ortì Inferiore (Reggio Calabria province), between the steps of a stairway, 38.147584°N–15.711343°E, 610 m a.s.l., *obs. et det.* V.L.A. Laface.
  Note. *O. stricta* grows in the crevices of the walls and sidewalks. It was probably introduced through nurseries or horticultural material. This species is naturalized or invasive in northern Italy and in some regions of central Italy, whereas it is reported as doubtful for Calabria [1]. Our record confirms its presence in the region.
(25) ***Passiflora caerulea* L.**
  Passifloraceae Neophyte Southern America Climbing phanerophyte
  First record for Calabria (casual)
  *Specimen*: 13 September 2019, SP1 Taurianova-Cittanova (Reggio Calabria province), roadside, climbed to a wire mesh, 38.352809°N–16.058705°E, 355 m a.s.l., *leg. et det.* V.L.A. Laface (REGGIO).
  Note. Species native to South America, introduced as an ornamental species together with numerous species of the genus *Passiflora* [93]. The only plant found grew by climbing on the wire mesh of an olive grove, very far from inhabited areas. The seeds were probably transported there by birds or small mammals as the fruits are very appetizing to wildlife. *P. caerulea* is reported as a casual alien in almost all regions of Italy (Abruzzo, Basilicata, Friuli Venezia Giulia, Lazio, Liguria, Lombardia, Marche, Molise, Puglia, Sardegna, Sicilia, Trentino-Alto Adige, Umbria, and Veneto). It is naturalized only in Toscana and Campania [1].
(26) ***Portulaca grandiflora* Hook.**
  Portulacaceae Neophyte Southern America Scapose therophyte
  First record for Calabria (casual)
  *Observata*: 29 July 2019, Via Mercato, Catona (Reggio Calabria province), roadside, 38.185898°N–15.638733°E, 4 m a.s.l., *obs. et det.* V.L.A. Laface; 25 July 2019, Via Nazionale, Catona (Reggio Calabria province), sidewalk, 38.178500°N–15.648642°E; 18 m a.s.l., *obs. et det.* V.L.A. Laface.
  Note. *P. grandiflora* cultivated for ornamental purposes in pots and flower beds produces very showy and colorful flowers. The plants observed grew in the crevices of the sidewalk. The species easily reproduces by seed, it is likely that the observed plants are derived from the seeds of pots and flowerbeds nearby. It was previously recorded as a casual alien in Emilia Romagna, Lazio, Liguria, Lombardia, Marche, Piemonte, Sardegna, Trentino Alto Adige, and Veneto, whereas it is naturalized only in Sicilia [1].
(27) ***Prunus armeniaca* L.**
  Rosaceae Archeophyte Culton Scapose phanerophyte
  First record for Calabria (casual)
  *Specimen*: 4 April 2019, Piani di San Nicola, Ortì (Reggio Calabria province), olive grove, 38.151968°N–15.695646°E, 560 m a.s.l., *leg. et det.* V.L.A. Laface (REGGIO).
  Note. *P. armeniaca* reproduces very easily by seed, often transported by man. It was not previously recorded as a casual alien species in Italy only for Valle d’Aosta, Puglia, and Friuli Venezia Giulia [1].
(28) ***Prunus dulcis* (Mill.) D.A. Webb** [≡*Amygdalus dulcis* Mill.]
  Rosaceae Archeophyte Feral Scapose phanerophyte
  First record for Calabria (casual)
  *Specimina*: 6 February 2020, C.da Fornace, Strada Gallina-Santa Venere (Reggio Calabria province), near an abandoned almond grove, 38.075486°N–15.724071°E, 491 m a.s.l., *leg. et det.* V.L.A. Laface (REGGIO); 13 September 2019, C.da Pietra, San Carlo (Reggio Calabria province), rocks emerging from the ground, 37.944144°N–15.864352°E, 173 m a.s.l., *leg. et det.* V.L.A. Laface (REGGIO); 13 September 2019, C.da Pietra, San Carlo (Reggio Calabria province), rocks emerging from the ground, 37.943588°N–15.864148°E, 172 m a.s.l., *leg. et det.* V.L.A. Laface (REGGIO); 13 September 2019, C.da Monte Callea (Reggio Calabria province), uncultivated field, 37.935116°N–15.922699°E, 76 m a.s.l., *leg. et det.* V.L.A. Laface (REGGIO); 19 May 2020, C.da Cambareri (Reggio Calabria province), roadside, 38.000372°N–15.676193°E, 357 m a.s.l., *leg. et det.* V.L.A. Laface (REGGIO); 19 May 2020 Motta San Giovanni (Reggio Calabria province) roadside, 37.999761°N–15.685197°E, 414 m a.s.l., leg. et det. V.L.A. Laface (REGGIO).
  *Observata*: 6 February 2020, Aretina Superiore (Reggio Calabria province), roadside, 38.068723°N–15.705494°E, 368 m a.s.l., *obs. et det.* V.L.A. Laface; 9 June 2020, Aretina Superiore (Reggio Calabria province), roadside, 38.099403°N–15.722838°E, 380 m a.s.l., *obs. et det.* V.L.A. Laface and CM Musarella.
  Note. Fruit tree cultivated mainly for the production of almonds, *P. dulcis* is used as food and in the confectionery industry. The species spreads rapidly by seed; all plants found were grown near almond groves nearby. According to Galasso et al. [1], this species is a casual alien throughout Italy excluding Lombardia, Valle d’Aosta, and Puglia where the species is naturalized.
(29) ***Salpichroa origanifolia* (Lam.) Baill.** [≡*Physalis origanifolia* Lam.]
  Solanaceae Neophyte Southern America Frutescent chamaephyte
  Change of status for Calabria: from naturalized to invasive (invasive)
  *Specimina*: 5 January 2020, Reggio Calabria (Reggio Calabria province), roadside, climbed to a wire mesh, 38.122064°N–15.664753°E, 59 m a.s.l., *leg.* G. Messineo, *det.* C.M. Musarella (REGGIO); 21 February 2020, Pentimele (Reggio Calabria province), roadside, climbed to a wire mesh, 38.142236°N–15.656332°E, 18 m a.s.l., *leg. et det.* C.M. Musarella (REGGIO); 4 March 2020, Catona (Reggio Calabria province), roadside, climbed to a wire mesh, 38.174193°N–15.649876°E, 27 m a.s.l., *leg. et det.* V.L.A. Laface (REGGIO); 3 March 2020, Gallico Inferiore (Reggio Calabria province), roadside, climbed to a wire mesh and on the wall of an old house, 38.166172°N–15.653259°E, 16 m a.s.l., *leg. et det.* V.L.A. Laface (REGGIO); 3 March 2020, Gallico Inferiore (Reggio Calabria province), climbed to a wire mesh and grow in uncultivated garden, 38.165846°N–15.652497°E, 14 m a.s.l., *leg. et det.* V.L.A. Laface (REGGIO); 3 March 2020, Gallico Inferiore (Reggio Calabria province) climbed to a wire mesh, 38.164773°N–15.650404°E, 9 m a.s.l., *leg. et det.* V.L.A. Laface (REGGIO); 3 March 2020, Fiumara Scaccioti (Reggio Calabria province), climbing on the bridge span, 38.156481°N–15.657227°E, 18 m a.s.l., *leg. et det.* V.L.A. Laface (REGGIO).
  *Observata*: 17 January 2020, Bolano, tra Catona e Villa San Giovanni (Reggio Calabria province), roadside, climbed to a wire mesh, 38.195878°N–15.636748°E, 15 m a.s.l., *obs. et det.* V.L.A. Laface; 10 February 2020, Marinella di Catona (Reggio Calabria province), roadside, climbed to a wire mesh, 38.192642°N–15.635087°E, 4 m a.s.l., *obs. et det.* V.L.A. Laface; 10 February 2020, Spontone (Reggio Calabria province), roadside, climbed to a wire mesh, 38.194584°N–15.635778°E, 5 m a.s.l., *obs. et det.* V.L.A. Laface.
  Note. Species already known for Calabria, it has recently been reported as naturalized for the region [49]. The plants observed often grow by climbing on wire mesh in very anthropized areas. In Italy, according to Galasso et al. [1], *S. origanifolia* is naturalized in Abruzzo, Calabria, Lazio, Marche, Piemonte, Puglia, Sicilia, Toscana, Umbria, invasive in Campania, and casual in Emilia-Romagna, Liguria, and Sardegna. For the several new stations found in which this species is so widespread and reproduces itself, we propose here the change of status from naturalized to invasive for Calabria.
(30) ***Sesbania punicea* (Cav.) Benth.** [≡*Piscidia punicea* Cav.]
  Fabaceae Neophyte Southern America Scapose phanerophyte
  Change of status for Calabria: from naturalized to invasive (invasive)
  *Specimen*: 21 August 2019, Lamezia Terme (Catanzaro province), 9 m a.s.l., in a water drainage channel near the airport, between the way and a cultivated field, 38.910900°N–16.252251°E, *leg. et det.* C.M. Musarella (REGGIO).
  Note. Recently reported as naturalized for Calabria in the province of Cosenza in 2015 [94], this is the second record for the region of *S. punicea*. This alien species shows a high degree of invasiveness in the new locality; it occupies more or less 100 m in length of a narrow water drainage channel along a road. The plants found were flowering and fruiting and several seedlings were also growing (Appendix B, Figure A9). For this reason, due to its rapid widespread and continuous reproduction, we propose the new status of invasive.
(31) ***Solanum tuberosum* L.**
  Solanaceae Neophyte Culton Scapose therophyte
  First record for Calabria (casual)
  *Specimen*: 11 January 2020, Sambatello (incrocio tra Via 25 Luglio e S.P.7) (Reggio Calabria province), roadside, water drainage channel, 38.179731°N–15.694848°E, 299 m a.s.l., *leg. et det.* V.L.A. Laface (REGGIO).
  Note. The sampled plant has probably escaped cultivation from nearby gardens or inadvertently been thrown by man and subsequently transported by water. *S. tuberosum* is reported for all Italian regions as a casual alien excluding Valle d’Aosta, Liguria, Basilicata, and Sicilia [1].
(32) ***Tecoma stans* (L.) Juss. ex Kunth** [≡*Bignonia stans* L.; *Stenolobium stans* (L.) D. Don; *Tecoma stans* (L.) Kunth var. *stans*; *Tecoma stans* (L.) Kunth var. *velutina* DC.]
  Bignoniaceae Neophyte Northern America Climbing phanerophyte
  First record for Europe (casual)
  *Specimen*: 19 October 2019, Svincolo Bocale - S.S. Jonica 106 (Reggio Calabria province), sidewalk, 37.987339°N–15.651794°E, 12 m a.s.l., *leg. et det.* V.L.A. Laface (REGGIO).
  Note. Native to southern USA, Mexico, the Caribbean, Peru, and Ecuador [93]. The dispersion occurs mainly through winged seeds dispersed by the wind, produced in large quantities almost all year round [95,96]. The plant observed probably originates from seeds of nearby houses, grown in the cracks of the sidewalk near a high traffic state road (Appendix B, Figure A10). Widely spread worldwide, *T. stans* is reported in Europe only for Malta as *T. stans* var. *sambucifolia* [97], whereas it is not given as present in Malta according to Euro+Med PlantBase [83]. Therefore, we consider this as the first report for Europe.
(33) ***Tradescantia sillamontana* Matuda**
  Commelinaceae Neophyte Southern America Rhizome geophyte
  First record for Calabria (casual)
  *Observata*: 1 December 2019, C.da Monte Callea (Reggio Calabria province), uncultivated field, 37.935131°N–15.922646°E, 76 m a.s.l., *obs. et det.* V.L.A. Laface (REGGIO).
  Note. Succulent species cultivated as an ornamental plant for its characteristic “hairy” leaves, it reproduces easily by vegetative way, and the plant probably escaped the cultivation from the nearby villas, after being thrown together with waste material (Appendix B, Figure A11). After the first record for Italy in Campania [98], this is the second one for the country and the first for Calabria.
(34) ***Washingtonia filifera* (Linden ex André) H. Wendl. ex de Bary** [≡*Pritchardia filifera* Linden ex André]
  Arecaceae Neophyte Northern America Scapose phanerophyte
  First record for Calabria (casual)
  *Specimen*: 10 January 2020, lungo la strada tra i dipartimenti di Agraria e di Ingegneria (Reggio Calabria province), sidewalk, 38.120043°N–15.666549°E, 85 m a.s.l., *leg. et det.* V.L.A. Laface (REGGIO).
  Note. Several individuals were found along a sidewalk. The seeds that are highly appetizing to birds are transported even at great distances. *W. filifera* has no particular ecological needs; it adapts easily to growing even in unfavorable conditions and is often found in the cracks in the walls and sidewalks. In Italy, based on Galasso et al. [1], the species is a casual alien in Abruzzo, Campania, Liguria, Puglia, Sardegna, and is naturalized in Sicilia. Recently, Lazzaro et al. [60] proposed this species for its inclusion on a national list of invasive species according to Regulation (EU) No. 1143/2014.
